# Supplementary material for: The Effects of AI-Guided Exercise and a Smart Ring on Arterial Stiffness: Protocol for a Randomized Controlled Trial
Source: JMIR Res Protoc. 2026 Aug 21;15:e98213. doi: 10.2196/98213 (PMC13495994; doi:10.2196/98213)
Supplement: Supplementary file 1 [file resprot-v15-e98213-s001.pdf]

Appendix 1: WHO Structured abstract

**Primary Registry and ID**	ClinicalTrials.gov: NCT06644014
**Date of Registration**	October 15, 2024
**Secondary ID(s)**	GONDOR-AS
**Source(s) of Support**	Oura Health Oy
**Primary Sponsor**	Oura Health Oy
**Secondary Sponsor(s)**	Kuopio Research Institute of Exercise Medicine (KuLTu)
**Contact for Public Queries**	Pauli Ohukainen (Oura Health Oy)
**Contact for Scientific Queries**	Pauli Ohukainen (Oura Health Oy); Heikki Pentikäinen (KuLTu)
**Public Title**	Reducing Arterial Stiffness with a Smart Ring and AI-Guided Exercise
**Scientific Title**	Reducing Arterial Stiffness with a Smart Ring and AI-Guided Exercise (GONDOR-AS): protocol for a randomized controlled trial
**Countries of Recruitment**	Finland
**Health Condition(s)**	Cardiovascular health, Arterial stiffness, Low cardiorespiratory fitness
**Intervention(s)**	1. AI-based Coaching: Personalized steady-state exercise guidance via Oura Advisor (LLM-based) . 2. HIIT: Supervised cycling. 3. Control: Oura Ring passive monitoring only.
**Inclusion/ Exclusion Criteria**	Inclusion: Ages 30-65; sedentary/low activity (<150min moderate or <75min vigorous/week). Exclusion: CAD, diabetes, uncontrolled hypertension, inability to wear a ring.
**Study Type**	Interventional; Randomized, parallel-group, 3-arm, blinded outcome assessment
**Date of First Enrollment**	October 16 2024
**Target Sample Size**	165
**Recruitment Status**	Ongoing
**Primary Outcome(s)**	Change in carotid-femoral Pulse Wave Velocity (cfPWV) and Change in VO2max (12 weeks)
**Key Secondary Outcome(s)**	Chance in Body weight, change in body fat, change in muscle mass (12 weeks)
**Ethics Review**	Regional Medical Research Ethics Committee of Eastern Finland Collaborative Area (Approved)
**Completion Date**	April 2026 (Estimated)
**Summary Results**	Not yet available (data collection and management ongoing)
**IPD Sharing Statement**	De-identified participant-level dataset and code to be made public via repository upon publication (pending specific participant permission)
